# Genetic Testing in Neurodevelopmental Disorders

**DOI:** 10.3389/fped.2021.526779

**Published:** 2021-02-19

**Authors:** Juliann M. Savatt, Scott M. Myers

**Affiliations:** Autism & Developmental Medicine Institute, Geisinger, Danville, PA, United States

**Keywords:** autism, intellectual disability, global developmental delay, genetic testing, neurodevelopment, exome sequencing, chromosomal microarray, fragile x

## Abstract

Neurodevelopmental disorders are the most prevalent chronic medical conditions encountered in pediatric primary care. In addition to identifying appropriate descriptive diagnoses and guiding families to evidence-based treatments and supports, comprehensive care for individuals with neurodevelopmental disorders includes a search for an underlying etiologic diagnosis, primarily through a genetic evaluation. Identification of an underlying genetic etiology can inform prognosis, clarify recurrence risk, shape clinical management, and direct patients and families to condition-specific resources and supports. Here we review the utility of genetic testing in patients with neurodevelopmental disorders and describe the three major testing modalities and their yields – chromosomal microarray, exome sequencing (with/without copy number variant calling), and *FMR1* CGG repeat analysis for fragile X syndrome. Given the diagnostic yield of genetic testing and the potential for clinical and personal utility, there is consensus that genetic testing should be offered to all patients with global developmental delay, intellectual disability, and/or autism spectrum disorder. Despite this recommendation, data suggest that a minority of children with autism spectrum disorder and intellectual disability have undergone genetic testing. To address this gap in care, we describe a structured but flexible approach to facilitate integration of genetic testing into clinical practice across pediatric specialties and discuss future considerations for genetic testing in neurodevelopmental disorders to prepare pediatric providers to care for patients with such diagnoses today and tomorrow.

## Neurodevelopmental Disorders

With a combined prevalence of ~17% among 3- to 17-year-old children in the United States, neurodevelopmental disabilities are the most prevalent chronic medical conditions encountered in pediatric primary care (1). The vast majority of these individuals have diagnoses captured within the *Diagnostic and Statistical Manual of Mental Disorders, 5*^*th*^ *Edition* (DSM-5) *neurodevelopmental disorders* category that includes intellectual disability (ID), global developmental delay (GDD), communication disorders (language disorder, speech sound disorder, childhood onset fluency disorder, and social/pragmatic communication disorder), autism spectrum disorder (ASD), attention deficit/hyperactivity disorder (ADHD), specific learning disorder (involving reading, written expression, and/or mathematics), and motor disorders (developmental coordination disorder, stereotypic movement disorder, and tic disorders) (2). Broader conceptualizations of neurodevelopmental disorders include conditions outside of the realm of the DSM-5, such as cerebral palsy (CP) and epilepsy, and sometimes neuropsychiatric disorders for which there is strong clinical and biological evidence of developmental origins, such as schizophrenia (3–6).

Neurodevelopmental disorders are characterized by developmental deficits in cognition, language, behavior, and/or motor skills that cause impairment of personal, social, academic, and/or occupational functioning (2, 7). These clinically and etiologically heterogeneous disorders represent manifestations of altered neural development and, as such, are typically diagnosed during infancy, childhood, or adolescence. Although subject to maturational changes, neurodevelopmental disorders are non-progressive and tend to follow a relatively steady trajectory rather than a pattern of remitting and relapsing (7). Tic disorders are an exception to the latter, since there is typically waxing and waning of core symptoms rather than a steady course. Because there is insufficient information to allow systematic classification of neurodevelopmental conditions based on etiology and pathophysiology, a descriptive (phenomenological) categorical nosology based on groups of signs and symptoms that define disorders or syndromes (e.g., ASD, ADHD, CP, etc.), has been adopted (8, 9). Descriptive categorical diagnoses are useful heuristics for improving interrater reliability and enhancing information exchange, but their limited alignment with many clinical and biological findings is well-described (8, 10–12). Pearn (9) noted that “syndromic diagnosis is a concise shorthand for describing a constellation of clinical symptoms and signs - but is an acknowledgment of causal ignorance, which in turn demands differential reappraisal as new biochemical, genetic, or ultimately molecular causes of the syndrome are defined.” Comprehensive developmental care, in addition to identifying appropriate descriptive diagnoses and guiding families to evidence-based treatments and supports, includes a search for an underlying etiologic diagnosis (13–16).

The scope of this review is limited to the genetic etiologic evaluation of children and adolescents with neurodevelopmental disorders. The focus is on individuals with diagnoses including GDD, ID, or ASD, for which there are existing guidelines for genetic testing, but we discuss the prospect of future genetic etiologic testing across broader neurodevelopmental disorders. Because of the focus on determining etiology using diagnostic genetic testing, the review does not cover pharmacogenomics, carrier testing, newborn screening, or pre-symptomatic/predictive testing. We provide an overview of the value of clinical genetic testing; the most commonly performed tests and the yield of these tests for determining a genetic diagnosis (focusing on established indications, ID/GDD and ASD); approaches to service delivery and implementation of genetic testing in clinical practice; and future directions, including emerging indications for genetic testing and forthcoming technologies.

## Utility of Genetic Testing in Individuals With Neurodevelopmental Disorders

Identification of an underlying genetic etiology for a child's categorical neurodevelopmental diagnosis (or diagnoses) can provide both clinical and personal utility to patients and their families. Table 1 provides several examples of genetic causes of neurodevelopmental disorders and the potential utility of identifying such a diagnosis. Establishing a genetic basis for a child's neurodevelopmental phenotype can provide additional information about their prognosis and enable caretakers to understand potential areas of need and opportunities for increased support (39). For example, a genetic diagnosis, and the prognostic information it provides, may facilitate acquisition of educational, disability, and employment services (40–45). Although currently infrequent in the case of neurodevelopmental disorders, a genetic diagnosis might also provide access to etiology-specific treatments (45–49). Given the number of treatments that are currently being explored in animal models and clinical trials, such gene-specific therapies are likely to become more ubiquitous (50). In the interim, as new therapies are being developed, a genetic diagnosis can provide access to condition-specific research protocols enrolling human subjects (51, 52). It is also important to note that elucidating etiologies and pathophysiologic processes is a necessary step toward developing animal models and human cell lines to use in pathogenesis studies, clinical trials, and, ultimately, mechanism-based targeted treatments for neurodevelopmental disorders (13, 53). In addition to facilitating medical management and access to services, a genetic diagnosis can also enable families to avoid unnecessary diagnostic tests (42, 45, 49, 52, 54) and, with an etiologic diagnosis in hand, families may be more empowered to avoid therapeutic interventions that are based on unfounded etiologic theories and are potentially harmful (e.g., chelation therapy) (55). Additionally, genetic testing can afford patients and their caregivers the ability to identify, treat, and/or prevent medical comorbidities at the time of diagnosis, as well as conditions that may develop later in life (42, 45, 49, 52, 56–65).

**Table 1:** Examples of clinical utility of specific genetic findings.

**Gene/copy number variant**	**Characteristics pertinent to clinical utility**	**Clinical utility**
15q11.2-q13.1 (BP2-BP3) interstitial duplication (17)	• Dup 15q syndrome, interstitial type • Dup 15q syndrome is caused by presence of at least one extra maternally derived copy of the Prader-Willi/Angelman critical region (PWACR) within chromosome 15q11.2-q13.1. • Approximately 20% of cases of dup 15q syndrome are due to an interstitial duplication on the maternally derived chromosome resulting in one extra copy of 15q11.2-q13.1 and, therefore, trisomy of this region. • Interstitial duplications are inherited from the mother in ~15% of cases and *de novo* in the other ~85%. • If the mother of a proband inherited a 15q interstitial duplication from her father, she will not have dup15q syndrome; she may appear to be unaffected or have milder features associated with paternal duplications.	• Genetic Counseling: Autosomal dominant inheritance with phenotype impacted by parent of origin due to methylation. Determining if the interstitial duplication was inherited or *de novo* can inform reproductive risks. Mothers who carry a 15q11.2-q13.1 interstitial duplication have a 50% chance of passing it on to each of their offspring.
17q12 deletion (18)	• 17q12 recurrent deletion syndrome; renal cysts and diabetes (RCAD) syndrome • Features include structural (e.g., renal cystic dysplasia), functional (e.g., tubulointerstitial disease) abnormalities of the kidneys and urinary tract, and maturity-onset diabetes of the young type 5 (MODY5).	• Genetic Counseling: Autosomal dominant inheritance and variants are usually *de novo*. Inheritance from a parent has been reported. Determining if the variant was *de novo* can provide information regarding reproductive risks for the family. • Periodic renal ultrasound, renal function tests, monitoring for tubulointerstitial disease, consultation with a nephrologist, and monitoring for MODY (including HgbA1C testing) are recommended.
*ALDH5A1* biallelic variants (19–21)	• Succinic semialdehyde dehydrogenase deficiency (SSADH deficiency) is a condition caused by biallelic pathogenic, loss of function variants in the *ALDH5A1* gene. • Biochemical phenotype includes elevated gamma-amino butyric acid (GABA) and gamma hydroxybutyric acid (GHB). • Features include age-dependent worsening of epilepsy severity, behavioral disturbances, sleep disturbances, and high risk of sudden unexpected death in epilepsy (SUDEP). Therapeutics that rescue a mouse model from premature lethality include GABA_B_ and GHB receptor antagonists, taurine, vigabatrin, ketogenic diet, and rapalog agents such as Torin 2 (an mTOR inhibitor).	• Genetic counseling: Autosomal recessive inheritance with recurrence risk of 25% for parents of an affected child. • Valproate is avoided when possible because of inhibition of potential residual enzymatic activity. • SGS742, a GABA_B_R antagonist, was the subject of a recently completed human clinical trial (results pending). • Additional opportunities for participation in clinical trials are anticipated.
*CACNA1C* (22)	• Timothy syndrome is caused by heterozygous, gain of function variants in the *CACNA1C* gene. • Features include long QT interval, other ECG abnormalities, congenital heart defects, frequent infections because of altered immune response, and intermittent hypoglycemia.	• Genetic counseling: Autosomal dominant and variants are typically *de novo*. Parental germline mosaicism has been observed. Determining if the variant was *de novo* can provide information regarding reproductive risks for the family. • Evaluation and treatment related to long QTc (beta-blocker, pacemaker, implantable defibrillator, close cardiac monitoring during anesthesia) and avoidance of drugs reported to prolong QT interval are recommended. • Avoidance/treatment of hypoglycemia is recommended.
*DHCR7 biallelic variants* (23, 24)	• Smith-Lemli-Opitz syndrome (SLOS) is caused by biallelic pathogenic, loss of function variants in the *DHCR7* gene, resulting in a deficiency of the enzyme 7-dehydrocholesterol reductase. • Biochemical phenotype includes elevated serum 7-dehydrocholesterol (7DHC) and, in most cases, low serum cholesterol. • Features include cleft palate, postaxial polydactyly, congenital heart defects, renal anomalies, cataracts, strabismus, photosensitivity, behavioral disturbances, and hypospadias in males.	• Genetic counseling: Autosomal recessive inheritance with recurrence risk of 25% for parents of an affected child. • Currently, most SLOS patients are treated with dietary cholesterol supplementation, despite limited evidence of efficacy. Efficacy of cholesterol supplementation is thought to be limited by the inability of dietary cholesterol to cross the blood-brain barrier, and a small controlled clinical trial did not observe differences in hyperactivity or problem behaviors between placebo and treatment phases. • Treatment with simvastatin can be considered. In a randomized, double-blind, placebo-controlled trial, treatment with simvastatin was associated with significant decreases in plasma dehydrocholesterol levels and improvement in irritability. • Treatment with haloperidol and perhaps other antipsychotic medications may exacerbate the biochemical sterol abnormalities in individuals with SLOS and cause an increase in symptoms, so they should be used with caution and avoided if possible. • Associated features require investigation or monitoring (e.g., cardiovascular and renal anomalies, cataracts, anticipatory guidance of photosensitivity).
*FMR1* (25–28)	• Fragile X syndrome is caused by loss of function of the *FMR1* gene. • 99% percent of cases of fragile X syndrome are caused by an expansion of the unstable CGG repeat sequence in the 5′ untranslated region (UTR) of the FMR1 gene. • Features include strabismus, seizures, musculoskeletal abnormalities (pes planus, pectus excavatum, scoliosis), cardiovascular phenotypes (mitral valve anomalies, aortic root dilatation), and behavioral disturbances.	• Genetic counseling: X-linked inheritance. All mothers of individuals with a *FMR1* full mutation are carriers of a premutation or full mutation. Reproductive risks are dependent on the mother's expansion size and, if the mother has a premutation, AGG interruptions within the CGG tract • Counseling and testing should be offered to extended family. In addition to reproductive risks, premutation carriers are at risk for fragile X-associated tremor/ataxia syndrome (FXTAS) and, in female premutation carriers, *FMR1*-related primary ovarian insufficiency. • Associated features require investigation or monitoring (e.g., scoliosis, cardiovascular phenotypes, seizures). • Several targeted pharmacologic therapies are under investigation (e.g., metformin, minocycline and lovastatin, sertraline).
*MECP2* (29)	• In females, *MECP2* disorders due to heterozygous, loss of function variants include Rett syndrome, variant Rett syndrome, and broader clinical phenotypes including mild intellectual disability. • In males, *MECP2* disorders due to hemizygous, loss of function variants include severe neonatal encephalopathy, pyramidal signs, parkinsonism, and macroorchidism (PPM-X) syndrome, as well as broader clinical phenotypes including syndromic and non-syndromic intellectual disability. • Features of individuals with *MECP2* disorders are variable but can include long QT interval, seizures, behavioral disturbances, breathing abnormalities, and scoliosis.	• Genetic counseling: X-linked inheritance and the majority of variants are *de novo*. Inheritance from a mildly or unaffected mother has been reported. Determining if the variant was *de novo* can provide information regarding reproductive risks for the family. • Evaluation and treatment related to long QTc (beta-blocker, pacemaker, implantable defibrillator, close cardiac monitoring during anesthesia) and avoidance of drugs reported to prolong QT interval are recommended. • Other associated features require investigation or monitoring (e.g., scoliosis, seizures, breathing abnormalities).
*PTEN* (30–32)	• *PTEN* hamartoma tumor syndrome is caused by heterozygous, loss of function variants in the *PTEN* gene. • Features include benign tumors (e.g., skin hamartomas, gastrointestinal polyps, thyroid nodules/goiter, fibrocystic breast disease, cerebellar dysplastic gangliocytoma), and increased risk for certain cancers (e.g., breast, thyroid, renal cell, endometrial, colorectal, and melanoma). • Expressivity is highly variable between and within families.	• Genetic counseling: Autosomal dominant inheritance with ~50–90% of pathogenic variants being inherited. • Determining if the variant was *de novo* or inherited can provide information regarding reproductive risks, cancer risks, and need for surveillance/management in family members. • Associated features require investigation or monitoring (e.g., breast, thyroid, renal cell, endometrial, colorectal, and skin cancers).
*SLC2A1* (33, 34)	• Glucose Transporter Type 1 Deficiency (GLUT1) deficiency is caused by heterozygous, loss of function variants in in the *SLC2A1* gene. • Features include epilepsy that tends to be unresponsive to antiepileptic mediations, acquired microcephaly, complex movement disorder. Milder phenotypes include the absence of seizures and paroxysmal events triggered by exercise, exertion, or fasting.	• Genetic counseling: Autosomal dominant in the majority of cases, with variants being *de novo* in 90% of cases. Rarely, autosomal recessive inheritance has been reported. Determining the inheritance within the family can provide information regarding reproductive risk and risk for phenotypic manifestations in family members. • Ketogenic diet to control seizures and improve gait disturbance.
*SYNGAP1* (35)	• *SYNGAP1-*Related Intellectual Disability is caused by heterozygous, loss of function variants in *SYNGAP1*. • Features include epilepsy, strabismus, musculoskeletal disorders (hip dysplasia, kyphoscoliosis, pes planus), constipation, and behavioral disturbances.	• Genetic counseling: Autosomal dominant and variants are typically *de novo*. Parental mosaicism (somatic and germline) has been reported. Determining if the variant was *de novo* can provide information regarding reproductive risks for the family. • Associated features requiring investigation or monitoring (seizures, kyphoscoliosis, hip dysplasia, constipation requiring intervention).
*TSC2/TSC1* (36–38)	• Tuberous sclerosis is caused by heterozygous, loss of function variants in *TSC1* or *TSC2*. Both genes are important for regulation of cell growth through the mTOR pathway. • Features include skin abnormalities (hypopigmented macules, angiofibromas, shagreen patches (connective tissue nevi), ungual fibromas), retinal hamartomas, renal manifestations (angiomyolipomas, cysts, renal cell carcinomas), subependymal nodules, cortical dysplasias, seizures, subependymal giant cell astrocytomas (SEGAs), rhabdomyomas, arrhythmias, lymphangioleiomyomatosis, multifocal micronodular pneumonocyte hyperplasia, and behavioral disturbances.	• Genetic counseling: Autosomal dominant inheritance and variants *de novo* in 2/3 of cases. Determining if the variant was *de novo* or inherited can provide information regarding reproductive risk, risk for phenotypic manifestations, and need for surveillance in family members. • Associated features requiring investigation or monitoring (e.g., CNS tumors, seizures, renal angiolyolipomas or cysts, cardiac rhabdomyomas, and arrhythmias). • Potential role for targeted pharmacologic therapy (mTOR inhibitors). Clinical trials examining the efficacy of mTOR inhibitors have shown evidence to support use of these therapies for SEGAs, renal angiomyolipoma, skin manifestations, and epilepsy.

Furthermore, genetic testing allows healthcare providers to refine recurrence risk counseling for the family to inform reproductive decision making (39, 42, 45, 49, 52, 58, 59, 63, 65–67). When an etiology is determined, the risk of recurrence for an individual family varies depending on the specific genomic variant(s) identified. For parents of a child with a neurodevelopmental disorder, recurrence may be 50%, for example, in the case of an inherited, maternally-derived chromosome 15q11–q13 interstitial duplication, 25% in the case of an autosomal recessive disorder such as Smith-Lemli-Opitz syndrome, or approximately the prevalence rate of the particular disorder in the general population (e.g., ~1.5% for ASD) if the child has a *de novo* explanatory variant. This refined recurrence risk information offers patients, parents, and family members an understanding of their reproductive risks and informs reproductive decision making, restores reproductive confidence, and enables prenatal diagnosis (45, 48, 68). Furthermore, an understanding of recurrence risk in subsequent children and generations can enable families and their medical providers to identify neurodevelopmental disorders and initiate beneficial behavioral treatments and therapies at earlier ages (69). In the case of inherited etiologies, identifying a genetic diagnosis for the proband may also provide diagnoses to other family members with a history of a neurodevelopmental disorder (70).

In addition to the clinical utility conferred through a genetic diagnosis, identifying an etiology can also provide psychosocial benefit to families. A genetic diagnosis can provide families with an explanation for their child's developmental history and, therefore, bring an end to the child's “diagnostic odyssey” that may have included years of uncertainty, anxiety, and evaluations (49, 58, 60, 71), and can also guide patients and families to condition-specific resources and supports (45). Receiving a diagnosis has also been shown to increase knowledge, provide a sense of empowerment (45), result in peace of mind (41), increase parental quality of life (72), decrease parental guilt (59, 73), and foster increased acceptance (74).

## Genetic Testing for Individuals With Neurodevelopmental Disorders

In addition to wanting to know their child's clinical diagnosis and prognosis, families typically want to know the cause of the child's developmental disability. In the last two decades, rapid advances in the development of genetic testing technologies and application of these technologies to well-characterized patient cohorts have revolutionized our ability to make specific genetic diagnoses in patients presenting with neurodevelopmental disorders. New genes are being implicated in neurodevelopment at a rapid pace. Online resources such as the Geisinger Developmental Brain Disorder Genes Database (https://dbd.geisingeradmi.org/), the Clinical Genome Resource's Gene Validity Curations and Dosage Sensitivity Map (https://search.clinicalgenome.org/kb/gene-validity/; https://dosage.clinicalgenome.org/), and DECIPHER's Development Disorder Genotype - Phenotype Database (https://decipher.sanger.ac.uk/ddd#ddgenes) can provide up to date information about genes' and genomic variants' relationship to neurodevelopmental disorders (75–78).

Genetic testing is now the standard of care for several neurodevelopmental disorders and the indications for testing will almost certainly broaden in the future. In practice, a genetic etiologic diagnosis may be suspected clinically and confirmed by gene-specific genetic testing or, more commonly, it may be revealed by chromosomal microarray (CMA) analysis, exome sequencing (ES), or *FMR1* CGG repeat analysis for fragile X syndrome completed as a routine part of the evaluation of a patient with ID/GDD or ASD in the absence of a clinically recognizable syndrome. It is important for pediatric clinicians to understand these common tests and their role in the care of children with neurodevelopmental disorders.

### Chromosomal Microarray

Genome-wide CMA has been endorsed as a first-tier test for several indications including in patients with ASD, ID, GDD, and/or multiple congenital anomalies (79, 80). CMA technologies (Comparative Genomic Hybridization (CGH) array CGH and Single Nucleotide Polymorphism (SNP)-based testing) detect copy number variants (CNVs) – gains or losses of chromosomal material (81). In some cases, such gains or losses affect gene function and impact health and development. The resolution, or size of gains and losses that can be detected by CMA, varies and is determined by the specific technology used and the genomic distance between DNA probes.

CNVs detected through CMA should be categorized into the following categories in accordance with the American College of Medical Genetics and Genomics (ACMG) and Clinical Genome Resource (ClinGen) guidelines (82).

1. *Pathogenic* – CNVs that are thought to be associated with disease. Pathogenic CNVs may include those that explain the patient's phenotype, those that are associated with carrier status for a recessive condition, and those that indicate disease risk for an unrelated phenotype. Pathogenic CNVs that explain a patient's neurodevelopmental history can include recurrent deletions or duplications such as 22q11.2 deletion syndrome or the 17p11.2 recurrent microdeletion that causes Smith-Magenis syndrome (83, 84), or novel gains or losses that impact dosage sensitive gene(s).
2. *Likely pathogenic* – CNVs that have considerable evidence to suggest that they are associated with disease but where additional evidence could further clarify the variant's pathogenicity.
3. *Uncertain Significance* – CNVs that do not have enough information to determine if they are pathogenic or benign. When a CNV with uncertain significance is identified, parental studies might be used to provide additional information to clarify pathogenicity.
4. *Likely Benign* – CNVs that have considerable evidence to suggest that they are not associated with disease but where additional evidence could further clarify this.
5. *Benign* – CNVs that are not thought to be associated with disease. These are often present in phenotypically normal individuals or in the general population.

Interpretations may change over time as new evidence emerges informing a variants' pathogenicity. Because CMA employs genome-wide testing, incidental findings, or those that are unrelated to the primary indication, may be identified (82). CNVs often include multiple genes and, in rare instances, a CNV might explain a child's neurodevelopmental history, but given the gene content, may also confer risk for an unrelated condition (61). In other cases, a CNV that is unrelated to a patient's phenotype but that has implications for care may be identified and reported through testing (85).

In addition to CNVs, laboratories that employ SNP-based CMA can also detect regions of homozygosity (chromosomal segments that are identical to one another). Identification of regions of homozygosity enables the potential detection of conditions that can be caused by uniparental isodisomy (UPD) such as Silver-Russell (maternal UPD chromosome 7), Angelman (paternal UPD chromosome 15), and Prader-Willi syndromes (maternal UPD chromosome 15) (86). In other instances, regions of homozygosity may be indicative of ancestral homozygosity or parental consanguinity (86–88). Regions of homozygosity can also be indicative of an increased risk for an autosomal recessive condition due to the potential for homozygous variation in single gene (88, 89).

Although CMA is able to detect CNVs, this technology has limited ability to detect balanced chromosomal rearrangements (translocations, inversions), trinucleotide repeat expansions, imbalances in the mitochondrial genome, epigenetic abnormalities (e.g., methylation abnormalities), sequence level variants, or low-level mosaicism for CNVs (Table 2) (65, 90). As stated above, the size of deletions and/or duplications that can be detected by CMA varies. Current clinical CMA platforms can detect CNVs ~400 kb in size (80) with many laboratories detecting those >250 kilobases. Certain regions may be more specifically targeted enabling even smaller CNV detection (Table 2).

**Table 2:** Genetic tests commonly used in evaluation of neurodevelopmental disorders.

**Test**	**Results/variants detected**	**Detection limitations**
Chromosomal Microarray (CMA)	• Copy number variants (generally >250 kb but could be smaller if region is specifically targeted) • Regions of homozygosity^*^	• Repetitive DNA sequences including trinucleotide repeat expansions (e.g., *FMR1* repeat expansion) • Balanced chromosomal rearrangements • Sequence level variants in the exome/genome • Mitochondrial variants • Epigenetics alterations (e.g., Methylation abnormalities, uniparental heterodisomy) • Low-level mosaicism
Exome Sequencing (ES)	• Sequence level variants in the coding region (exome) • Copy number variants^**^	• Repetitive DNA sequences including trinucleotide repeat expansions (e.g., *FMR1* repeat expansion) • Balanced chromosomal rearrangements • Smaller copy number variants including deletions/duplications involving one to two exons • Mitochondrial variants • Epigenetics alterations (e.g., Methylation abnormalities) • Intronic/non-coding variants • Variants in regions of the exome that are not well-covered by sequencing
*FMR1* CGG Repeat Testing	• CGG repeat number in the *FMR1* gene	• Sequence level variants in *FMR1* or elsewhere in the exome/genome • Copy number variants • Balanced chromosomal rearrangements • Exon-level deletions/duplications • Mitochondrial variants • Epigenetics alterations (e.g., Methylation abnormalities)

* - SNP Based CMA;

** *- Several laboratories are now calling CNVs as a routine part of ES and this trend will continue to expand*.

*Sources: Miller et al. (80); Srivastava et al. (65); Monaghan et al. (25); Collins et al. (26, 27)*.

Additional testing discussed in this review can aid in detection of some variants that CMA is unable to detect, including *FMR1* CGG repeat analysis for the trinucleotide repeat expansion that causes fragile X syndrome and ES for detection of exonic sequence variants. Cytogenetic testing, including G-banded karyotype and/or florescence *in situ* hybridization (FISH) as well as methylation testing, can detect additional variants or clarify results from CMA. As reviewed elsewhere, such testing should be considered on a case by case basis (e.g., for detection of balanced rearrangements and other complex rearrangements or mosaicism for partial or whole chromosome aneuploidy, or for clarification of the location of a duplication) (90).

#### Diagnostic Yield of CMA in ID/GDD and ASD

Authors reporting CMA yield use variable nomenclature to describe variants' pathogenicity and diverse interpretation practices, thus limiting between-study comparisons and pooling of data across studies of CMA diagnostic yield. The diagnostic yield of CMA is the proportion of tests performed that identify a variant that is considered causative for a patient's phenotype. Taken together, a causative result can be identified by CMA in 15–20% of individuals with ID/DD, ASD, and/or multiple congenital anomalies (54, 79, 80). For the purpose of trying to capture yield specific to neurodevelopmental disorders, one can examine 19 studies that report on the yield of CMA in more than 150 individuals with ID/GDD where the diagnostic yield ranged from 4.5 to 28.0% (median 13.7%) (91–109). Additionally, one can look to 11 studies limited to patients with ASD (each with a sample size ≥50), in which CMA identified a causative variant in 1.5 to 20.5% of subjects (median 8.1%) (96, 98, 99, 104, 109–115). Because karyotype was the standard etiologic approach before the clinical integration of CMA, many publications summarizing diagnostic yields of CMA excluded patients with an abnormal karyotype. Since the majority of pathogenic results identified by karyotype would be detected by CMA, a number of publications may be reporting yields ~3.7% percent lower, on average, than if CMA had been applied as a first-tier evaluation (116). Overall, the diagnostic yield of CMA in patients with ID, GDD, and/or ASD suggests that CNVs comprise a substantial proportion of underlying genetic etiologies.

Although the diagnostic yield of CMA has been well-examined in the literature, there are few studies exploring the frequency that incidental findings are identified with this testing technology. In two studies, 0.15–0.48% of patients had a CNV that included a gene associated with hereditary cancer pre-disposition (117, 118), and in a third study, 1.2% had a CNV that included a gene associated with an adult-onset condition (119) The pathogenicity of these CNVs were not reported by the study authors, so the true rate of pathogenic incidental findings in these study populations is somewhat unclear (117–119). Taken together, however, these studies suggest that clinically significant incidental findings likely occur in <1% of patients undergoing CMA.

### Exome Sequencing

In the last decade, the arrival of high-throughput sequencing technologies, collectively referred to as next generation sequencing (NGS) or massively parallel sequencing, has reduced the cost and increased the speed of sequencing, enabling laboratories to sequence large amounts of DNA. Rather than testing a single gene or several genes, laboratories can now offer sequencing of extensive gene panels, the exome, or the genome.

In ES, protein coding regions, or exons, of the genome are sequenced. The exome makes up ~1.5–2% of the genome, but the vast majority of alleles underlying Mendelian disorders impact coding sequences (120). ES allows detection of sequence-level, single gene variants across almost all of the exome and a small number of intronic nucleotides at the boundaries of each exon. In addition to sequence-level variant detection, several clinical laboratories are now incorporating CNV calling into ES and are able to detect multi-exon deletions and duplications (65, 121, 122) and more laboratories will likely add this to their exome analyses moving forward. Shorter CNVs including those that involve one to two exons are less reliabily detected using ES (121). Given the breadth of information that is generated using ES, parental samples are recommended for analysis (ideally trio analysis or duo analysis if only one parent is available) since this can reduce the number of candidate variants that require review and facilitate variant interpretation (78, 123).

As with CMA, variants detected by ES are categorized as pathogenic, likely pathogenic, uncertain significance, likely benign, or benign in accordance with ACMG/ Association for Molecular Pathology (AMP) sequence variant interpretation guidelines (123). Due to the rapid increase in our understanding of genes and genomic variants, improved variant annotation and filtering, and evolving patient phenotypes (65, 124–129), an iterative reanalysis of ES data may enable additional diagnoses. Over time, a variant's pathogenicity may be updated as new information emerges. ES also includes analysis of genes not yet associated with a specific phenotype. As a result, iterative reanalysis might also identify a novel variant.

ES, like CMA, may identify results that are clinically relevant but unrelated to the patient's neurodevelopmental disorder. ES includes almost all of the coding portions of known genes, providing an opportunity to examine sequence data for pathogenic/likely pathogenic variants in medically actionable genes that are unrelated to the indication for testing (secondary findings). The ACMG has recommended that clinicians notify their patient if a variant known or expected to increase disease risk was identified in a list of, currently 59, clinically actionable genes (130, 131). ACMG has specified that patients and families have the ability to opt out of such findings (132) and will continue to update this list of clinically actionable genes as additional evidence emerges (130). In addition to secondary findings, the use of parental samples in ES can result in incidental identification of possible misattributed parentage; reporting and disclosure of this information varies between laboratories and clinicians (133, 134).

Although ES captures the majority of the exome, the exome is not covered in its entirety and coverage may differ across platforms and laboratories (135). As a result, ES may not detect all coding, sequence variants. ES also has lower sensitivity for detection of mosaicism and exon-level deletions and/or duplications compared to gene panels that include deletion/duplication analysis (65). An increasing number of laboratories are calling CNVs from ES data; laboratories that are detecting and reporting CNVs have limited ability to detect deletions or duplications involving only one to two exons, however (Table 2) (121). Furthermore, ES is unable to detect repetitive DNA sequences including trinucleotide repeats (e.g., the CGG repeat expansion that causes fragile X syndrome), intronic/non-coding variants, mitochondrial variants, epigenetic variants (e.g., methylation abnormalities), or balanced chromosomal rearrangements.

#### Diagnostic Yield of ES in ID/GDD and ASD

The diagnostic yield is the proportion of exome analyses with variants that are determined to be pathogenic or likely pathogenic and explain the patient's phenotype. A recent meta-analysis of 21 ES studies that focused on isolated neurodevelopmental disorders (GDD, ID, and/or ASD; *n* = 3,173) identified a diagnostic yield of 31% (95% CI 25–38%) (65). When nine additional studies of individuals with these neurodevelopmental disorders plus associated neurological or syndromic conditions or clinical characteristics were included, increasing the total number of participants to 3,350, the yield was 36% (95% CI 30–43%). In a large, laboratory-based study not included in the meta-analysis because the cohort included a potentially broader group of neurodevelopmental phenotypes, the diagnostic yield among individuals undergoing ES due to neurodevelopmental disorders (ID, ASD, developmental delay, or speech delay) with or without involvement of other organ systems was 25.4% (425 of 1,673) (136).

Delineation of the diagnostic yield of ES for cohorts ascertained for ID/GDD vs. ASD is limited by the phenotype data reported in the literature. Srivastava et al. (65) reported in their meta-analysis that among studies including individuals with primarily ID (*n* = 10), the diagnostic yield was 39% (95% CI 29–50%), whereas studies including individuals with primarily ASD (*n* = 5) had a yield of 16% (95% CI 11–24%) and those with a more heterogenous mix of ID and/or ASD (*n* = 6) identified a diagnosis in 37% of participants (95% CI 29–46%) (65). Among seven ES studies each involving more than 50 individuals with ID/GDD, a diagnosis was established in 34% of patients (range 28–43%) (128, 137–142). Studies restricted to clinically ascertained samples of patients with ASD and analyses of ASD subgroups within the large, laboratory-based samples have reported lower yields of 8–26% (median 15%) (139, 140, 143–146). There is substantial variability among these studies regarding the information provided about cognitive status and the stringency of ASD diagnosis. In most cases the patients included had previously had genetic testing including fragile X analysis and CMA that did not result in a molecular diagnosis. ES reveals two or, rarely, three molecular diagnoses in ~1% of individuals undergoing clinical testing (136, 140). When this occurs, the patient typically has a “blended phenotype,” with features that are accounted for by each of the pathogenic variants and not by a single molecular diagnosis.

Several studies suggest that the diagnostic yield of ES is higher when trios (probands and parents) are tested than when only the DNA of probands is sequenced (139, 140, 147). Because of the rapid advances in gene and variant curation and factors such as evolution of the phenotype in an individual over time, periodic reanalysis of ES data may enhance the diagnostic yield considerably (48, 124, 127, 138, 148, 149). There is wide variability in the rate of secondary findings among individuals who undergo ES; several recent large studies have reported rates of 2–6% (136, 140, 150, 151). These reportable secondary findings, which are most commonly related to cardiomyopathies, cardiac conduction disorders, hereditary cancer pre-disposition, or familial hypercholesterolemia, often result in additional testing and/or surveillance of the proband and relatives (140).

#### Exome Sequencing Compared to More Targeted Sequencing

In addition to ES, single gene testing and targeted next-generation sequencing panels have been used in evaluation of patients with neurodevelopmental disorders including ID, GDD, and ASD historically, and a number of clinical laboratories currently offer such testing. Compared to ES, more targeted sequencing allows for increased read depth and sequence coverage thus increasing detection of mosaicism (152). Moreover, single gene testing and gene panels are better able to detect indels and those that include deletion/duplication analysis are better able to detect exon-level deletions and/or duplications (65, 153). While ES identifies 100–200 potentially deleterious sequence variants on average, more targeted analysis identifies a smaller number of variants requiring less analysis and reducing the potential for variants of uncertain significance (81, 120, 154).

Although there are some advantages to more targeted testing and clinical scenarios that warrant such testing, the genetic heterogeneity of ID, GDD, and ASD and frequency of causative *de novo* variants, ES is an appropriate approach for many patients with these diagnoses (129). Studies have found that ES can detect more than 98% of pathogenic variants identified on gene panels (155). Additionally, the gene content of targeted gene panels vary significantly between laboratories meaning that diagnostic yields also vary. In Hoang et al. (156), comparison of ASD gene panels across 21 laboratories found that the number of genes included on ASD-related panels ranged from 11–2,562 and only a single gene (*MECP2*) was included on all panels. In a simulation study comparing ES to gene panels, providers were asked to choose a commercially available gene panel as an alternative when ordering ES for a patient; of the patients receiving a diagnosis through ES, 23% would not have had their variant identified through the provider-chosen gene panel (157). Additionally, as an increasing number of genes are being implicated in Mendelian conditions (158) including neurodevelopmental disorders, panels quickly can become out of date and updating panels can be a time-consuming process (159). After undergoing gene panel testing, 11% of panel-negative cases in one cohort received a diagnosis via ES; many of these diagnoses were attributed to a gene-disease relationship being identified after the panel assay was established (154). In addition to being able to more readily report recently described genes implicated in neurodevelopmental disorders, ES also enables increasing diagnoses over time due to reanalysis. Unlike gene panels, ES includes analysis of genes not yet associated with a specific phenotype. As a result, iterative reanalysis enables reporting of novel variants recently implicated in neurodevelopment and can allow for diagnoses into the future while gene panels typically only enable reanalysis of the sequenced genes. Finally, more targeted testing has been viewed as a less expensive testing option; however, more recent publications have suggested that ES is cost-effective (142, 157, 160–163).

### Variants of Uncertain Significance (VUS) From CMA and ES

Although ES and CMA have revolutionized our ability to detect genomic variants, there are limitations in our ability to interpret such variants and determine their impact on health and development. Copy number and sequence variants identified through CMA and ES might be interpreted as variants of uncertain significance (VUS) based on available evidence. VUS include variants in known disease genes that have insufficient evidence to be classified as benign or pathogenic as well as sequence and copy number variants that involve genes that are not yet associated with a disease or phenotype (also referred to as genes of uncertain significance/ candidate genes) (82, 123). VUS in patients undergoing CMA due to a history of developmental delay, ID, ASD, and/or multiple congenital anomalies have been reported to carry at least one VUS 7.9–19% of the time (94–96, 104, 105, 107, 164). The frequency of VUS in patients undergoing ES depends on a number of factors including the reporting laboratory's reporting practices (165), phenotypic features provided by the ordering clinician (166), and inclusion of parental samples. Several studies examined VUS rates among patients undergoing ES for a variety of indications and found VUS rates of 25.3–86% (63, 166, 167).

Uncertain results, although not unique to genetics (168), can pose challenges for patients and providers alike. Compared to other potential results, providers are least comfortable explaining VUS results to families and report that additional preparation is required for such results (169–171). Non-genetics providers express a need for additional education and access to genetics professionals' expertise to facilitate understanding and disclosure of such results (169). Despite expressing less comfort with VUS results and calls for more support and education, non-genetics, pediatric providers that regularly order testing appear to be able to understand and interpret VUS results accurately (172).

Studies of parents have suggested that they are interested in receiving uncertain results and those that receive such results post-natally see the them as important, often demonstrate an understanding that the result is uncertain, and acknowledge that future advancements can increase understanding (73, 171, 173–178). Although parents seem to accurately recall the concept of uncertainty, parents report difficulty understanding how the result impacts them and their child, and some over interpret the variant as being causative (73, 170, 173, 174). Emotional reactions to VUS vary between families and over time (174, 176).

Given the potential for misunderstandings and variable emotional reactions, providers need to be equipped to discuss uncertain results or refer on to providers with genetics expertise (73). The option for in-person, timely discussions with empathic and honest providers and access to supplemental information including documentation of the result have been suggested to improve understanding of VUS (73, 173, 176, 177). In contrast, internet searches have been shown to increase uncertainty; therefore, anticipatory guidance about misleading or irrelevant online information should be provided to families receiving VUS results (73). In addition to providing families with appropriate information, support, and resources to facilitate understanding of uncertain results, providers returning VUS should consider if additional evaluations that could inform the pathogenicity of the variant are indicated such as parental or familial testing, imaging, or specialist referrals (82, 123, 137, 179). Furthermore, knowledge of genomic variants and their relationship to health and development will continue to improve. As such, VUS will be updated to benign or pathogenic over time. For example, in one study of 2,250 patients undergoing ES reanalysis, 23 had variants initially reported as VUS upgraded to diagnostic (125). Patients receiving VUS results need to be informed of the potential for interpretation updates and ordering providers should discuss the process for reassessing variants.

### *FMR1* CGG Repeat Analysis

Fragile X syndrome, caused by loss of function of the *FMR1* gene, is thought to be one of the most common inherited genetic causes of ID and ASD (54, 180, 181). Ninety-nine percent of cases of fragile X syndrome are caused by an expansion of the unstable CGG repeat sequence in the 5′ untranslated region (UTR) of the *FMR1* gene (25–27). Most individuals in the general population have 44 or fewer CGG repeats, while more than 200 CGG repeats in the *FMR1* gene results in hypermethylation and, consequently, transcriptional silencing of the gene (182, 183). The *FMR1* gene is located on the X chromosome (Xq27.3) and, consequently, mutations can cause variable phenotypes in males and females (54, 184–186). *FMR1* CGG repeat analysis is usually completed using polymerase chain reaction (PCR) analysis and Southern Blot Analysis (25). *FMR1* CGG repeat analysis can yield four main categories of results (25):

1. *Normal:* ≤44 repeats.
2. *Intermediate (Inconclusive, Borderline, Gray Zone*): 45–54 repeats. Alleles with 45–54 repeats have not been observed to expand to a full mutation in one generation. Because minor increases or decreases in repeat size can occur, alleles of this size could be associated with fragile X syndrome in future generations.
3. *Pre-mutation:* 55–200 repeats. Expansions in *FMR1* of this size are unstably passed from parent to child and, when passed from the mother, expansion from the pre-mutation to a full mutation may occur. The risk for expansion is greatest in those with larger repeat sizes (187). Furthermore, the presence of AGG interruptions within the CGG repeat tract is associated with decreased risk for expansion (187–189).
4. *Full Mutation*: >200 repeats (typically several hundred to several thousand repeats).

In addition to the risk of expansion to a full mutation in offspring, female pre-mutation carriers are at risk for hypergonadotropic hypogonadism (fragile X pre-mature ovarian insufficiency) before age 40 years. Although more prevalent in males, both male and female pre-mutation carriers are also at an increased risk for fragile X-Associated Tremor/Ataxia Syndrome (FXTAS), a late-onset neurodegenerative condition characterized by cerebellar ataxia, intention tremor, cognitive impairment with increased penetrance observed in males (190). When offering *FMR1* CGG repeat testing, pre-mutation carrier status may be identified in the proband and/or relatives and incidentally identify risks for these adult-onset conditions (181).

*FMR1* CGG repeat testing cannot detect other variants causing loss of function of the *FMR1* gene (e.g., deletions, sequence variants that result in protein truncation), which are rare. Testing does not detect other genomic causes of neurodevelopmental disorders (e.g., sequence variants in other genes, copy number variants, epigenetic abnormalities) (Table 2).

#### Diagnostic Yield of *FMR1* CGG Repeat Analysis in ID/GDD and ASD

The yield of fragile X testing among individuals with ID/GDD and/or ASD varies widely based on study design and characteristics of the population being tested, such as severity of cognitive impairment, whether both males and females are included, and whether clinical judgement or phenotypic feature checklists were used to exclude some potential participants (191, 192). For example, the diagnostic yield varied from 0.5 to 6% among 14 studies reviewed by Peprah (192) that included at least 200 individuals with ID who were ascertained either through clinical referral or special needs service utilization (192). Hunter et al. (191) identified 15 studies that estimated the frequency of the full mutation in populations with ID before extrapolating to the total population, with the goal of including them in a meta-analysis, but it was not possible to combine the data and calculate a valid mean prevalence due to variability in study methods and lack of reported measures of uncertainty (191).

Several studies that attempted to capture populations of males with neurodevelopmental disorders for assessment of the rate of fragile X syndrome reported full mutations in 8/611 (1.3%) with unexplained ID, 20/3,738 (0.5%) with special education needs related to cognitive deficiencies, and 7/2,471 (0.28%) receiving special education services (excluding isolated speech therapy or gifted services) (193–195). An epidemiological study of a representative sample of 3,313 people with ID (56% male) in the Netherlands included 1,143 individuals (55% male) who were not eligible for testing because they had other etiologic diagnoses, including fragile X syndrome (30 males and 2 females; 4.75 and 0.39%, respectively), or had previous negative clinical testing, and 2,170 (57% male) who were eligible for testing as part of the study (196). Among those who were eligible, 1,520 individuals (57% male) consented and were tested. Full mutations were identified in 9/866 males (1.0%) and 2/654 females (0.31%) (196). Allowing the unknown diagnostic yield for those who were eligible but did not consent to testing to vary from 0.5 to 2.0 times the sex-specific diagnostic yield of those who were tested and including the ineligible individuals, the overall prevalence estimates are 2.2–2.5% for males with ID and 1.3–1.6% for females with ID. The rates of full *FMR1* mutations identified among clinical ASD cohorts are generally lower than those identified in clinical ID cohorts. Although early studies suggested higher rates, the larger studies published in the last decade (*n* = 142 to *n* = 861) have identified fragile X full mutations in only 0.23 to 1.2% of individuals ascertained for ASD diagnosis (111, 197–201). The combined yield of these studies was nine full mutations in 1,984 individuals tested (0.45%), including seven males and two females. Fragile X syndrome has been identified in females with ASD with and without ID (202–204).

The diagnostic yield of fragile X testing has also been described by several clinical laboratories, typically with little information available about phenotype. For example, a large-volume commercial diagnostic laboratory reported *FMR1* full mutation alleles in 1.4% of 59,707 males and 0.61% of 59,525 females tested post-natally over a 14-year period (1992–2006) (205). More recently, two university hospital diagnostic laboratories reported yields of 0% (0/654) and 0.9% (11/1,177) in males under age 22 and 19 years, respectively (206, 207). A similar yield of 0.8% (43/5,401) was reported by another hospital-based genetics laboratory that did not describe the diagnostic rate in males and females separately (208). Borch et al. (209) found a diagnostic yield of 1.2% (30/2,486) among pediatric patients who had fragile X testing, including 1.3% of males (25/1,919) and 0.9% of females (5/567). The vast majority, 96% (29/30), had clinical features and/or family history that were suggestive of fragile X syndrome.

### Additional Factors Influencing the Diagnostic Yield of CMA, ES, and *FMR1* CGG Repeat Analysis

Although studies vary in the level of phenotypic detail provided, several authors reporting on yields of CMA, ES, and *FMR1* CGG repeat analysis in patients with neurodevelopmental disorders suggest that specific characteristics and additional diagnoses are associated with increased diagnostic yields (95, 111, 113, 140, 146, 198, 201, 210, 211). Lower IQ, dysmorphic features, and congenital anomalies have been found to be associated with higher diagnostic yield of CMA and ES in some studies examining yield in patients with ASD (111, 113, 146, 198, 201). Similarly, in Fan et al. (95), co-occurring congenital heart defects, facial dysmorphisms, microcephaly, and hypotonia were associated with increased diagnostic yield of CMA in patients with developmental delay or ID (95). A higher diagnostic yield has also been reported for moderate to severe ID than for mild ID. Among individuals with severe ID, it is estimated that more than 60% harbor causative CNVs or exonic sequence variants (211, 212). Finally, in a meta-analysis of published studies using clinical checklists among patients with ID undergoing testing for fragile X, soft and velvety skin on the palms with redundancy on the dorsum of the hand, large prominent ears, pale blue eyes, family history of ID, autistic behavior, flat feet, and plantar crease all are correlated with an increased likelihood of identifying a *FMR1* full mutation (210). Although specific features and additional diagnoses have been correlated with increased diagnostic yield, the yield in patients with isolated ID/GDD and/or ASD is high enough to warrant an etiologic genetic investigation following the application of these categorical diagnoses.

### Summary of Diagnostic Yield

Although heterogeneous study designs and methodologies limit conclusions that can be drawn regarding diagnosis-specific diagnostic yields, current evidence suggests that among patients with ID or GDD, the diagnostic yields are at least 15% for CMA, 35% for ES, and 1% (in females) to 2% (in males) for *FMR1* CGG repeat analysis. For those with primarily ASD, the diagnostic yields are at least 10% for CMA and 15% for ES, and 0.5% or less for *FMR1* CGG repeat analysis. Across all of these tests and clinical diagnoses, the diagnostic yield rates are higher in the presence of lower IQ, neurological comorbidities, dysmorphic features, and congenital anomalies. The cumulative diagnostic yield for all three tests, therefore, is over 50% for individuals with ID/GDD and over 25% for individuals with primarily ASD.

## Approach to Genetic Etiologic Evaluation

Due to the yields of genetic testing and corresponding potential for clinical and personal utility, there is consensus that genetic testing should be offered to patients with GDD, ID, and/or ASD diagnoses (13–15, 65, 80, 213). The specific testing algorithm varies between guidelines and consensus statements (214), and many recommendations predate the broad integration of exome sequencing into clinical care (Table 3).

**Table 3:** Genetic testing guidelines for ID/GDD and/or ASD.

**Organization**	**Recommendation(s) for genetic testing**
Autism and Intellectual Disability Committee of the American Academy of Child and Adolescent Psychiatry (AACAP) (15)	• CMA in all individuals with ID/GDD and/or ASD • *FMR1* repeat analysis in males and females with ID or a family history of ID • Depending on history and physical examination, consider: °*PTEN* testing if head circumference (HCM) is more than 2.5 SD above the mean for age in a child with ID/GDD and/or ASD °*MECP2* testing for Rett syndrome in females with severe ID ° Karyotype if a chromosomal syndrome is suspected • If other investigations do not provide an etiology and there are unresolved clinical findings, consider ES and mitochondrial DNA testing.
American Academy of Pediatrics (AAP) (13, 14)	• If a comprehensive history and exam are indicative of a specific syndrome or disorder, proceed with specific testing in patients with ASD and/or ID/GDD. • In all individuals with ASD and ID/GDD without specific findings, consider CMA and *FMR1* CGG repeat analysis. • In females with ASD and/or ID/GDD without specific findings, consider *MECP2* testing. • In males with ID/GDD without specific findings, consider an X-linked ID panel (XLID). • If an etiology is not identified, consider a referral to genetics for additional work-up including possible ES.
International Standard Cytogenomic Array (ISCA) Consortium (80)	• CMA is the first-tier genetic test in patients with GDD/ID, ASD, and/or multiple congenital anomalies.
American College of Medical Genetics (ACMG) (54)	• After a detailed family history and physical examination, proceed with specific testing for patients with ASD if a syndrome is suspected or if features are suggestive of a mitochondrial or metabolic condition. • If family history and physical exam are not suggestive of a specific diagnosis, metabolic, or mitochondrial condition, proceed with CMA for all patients with ASD and *FMR1* repeat analysis for all males with ASD. • If CMA (in males and females) and *FMR1* repeat analysis (in males) are not diagnostic, consider: °*MECP2* sequencing in all females with ASD °*MECP2* duplication testing in males with ASD if phenotype is suggestive °*PTEN* testing in patients with ASD if HCM is more than 2.5 SD above the mean for age °*FMR1* repeat analysis in females with ASD and additional features suggestive of fragile X (e.g., family history and phenotype)
American College of Medical Genetics (ACMG) (79)	• CMA is the first-tier genetic test in patients with multiple congenital anomalies that are not indicative of a specific genetic syndrome and those with non-syndromic ID/GDD, and ASD.
Multidisciplinary Expert Consensus Panel (65)	• ES in all individuals with ID and/or ASD • If ES is non-diagnostic and does not including copy number variant analysis, proceed with CMA. • If ES (and CMA if needed) is non-diagnostic, reanalysis of data from testing should be undertaken periodically.
American Academy of Neurology (AAN) and Child Neurology Society (CNS) (213, 215)	• High resolution karyotype and *FMR1* repeat analysis for patients with ASD that also have ID, family history of fragile X and/or ID, or dysmorphic features.
	• After obtaining a detailed medical, developmental, and family history for patients with ID/GDD, if a specific etiology is considered, perform appropriate testing such as single gene testing, metabolic testing, or XLID panel. • If a specific etiology is not suspected, perform CMA (or, if not possible, karyotype and subtelomeric FISH) for all individuals with ID/GDD, *MECP2* testing for females with moderate to severe ID/GDD, and *FMR1* repeat analysis in all individuals with mild ID/GDD. • If these and other etiologic work-ups are negative, consider a referral to genetics.

Despite widespread recommendations for genetic testing for neurodevelopmental disorders in pediatric patients, surveys, interviews (69, 214, 216–221) and retrospective studies of clinical cohorts (111, 198) suggest that that a minority of children with ASD (16.5%-45%) and ID (43%) in the United States have undergone any genetic testing. Furthermore, a study examining pediatric providers' use of genetic testing in the care of simulated patients with ID, GDD, and/or ASD suggest that genetic testing is underutilized (222). In Europe, rates of testing vary from country to country with 61.7% French parents reporting that their child with ASD underwent some diagnostic genetic test compared to 13% of children with ASD in Spain (216, 223). Although there is consensus agreement that a genetic evaluation should be offered to individuals with GDD, ID, and/or ASD, this does not appear to be occurring in practice. Pediatric providers caring for patients with neurodevelopmental disorders should work to address this gap in care.

### Directly Consent for and Order Testing

Pediatric providers, from general practitioners to neurodevelopmental pediatricians, are in a position to empower themselves to consent for and order genetic testing. Given the increasing availability of genetic testing and limited number of genetics professionals, genetics providers cannot and need not be involved in all instances of consent for genetic testing and disclosure of results (224–227). Genetic testing for patients with neurodevelopmental disorders has shifted to be within the purview of non-genetics providers and is something that pediatric providers can be equipped to undertake (214, 228–233). Below we describe a structured but flexible approach to facilitate integration of genetic testing into clinical practice across pediatric specialties (Table 4).

**Table 4:** Approach to genetic etiologic evaluation in GDD, ID, and/or ASD.

**1. Complete physical examination and collect developmental, medical, and family history.** • If a specific etiology is suspected, pursue the appropriate specific genetic testing (e.g., *PTEN* sequencing and deletion/duplication analysis, *MECP2* sequencing and deletion/duplication analysis, *FMR1* CGG repeat analysis) • If a specific etiology is not suspected, proceed to step 2. **2. Pursue broad examination for causal exonic sequence variants and copy number variants.** • This is most efficiently achieved through ES with CNV calling. • If insurance restrictions or laboratory offerings dictate a stepwise approach to CNV analysis and ES, CMA and ES can be pursued individually. ES should be pursued as the initial evaluation when possible. • ES should be performed using a trio analysis when possible to decrease the number of candidate variants and inform variant classification. • If an etiology is not determined, proceed to step 3. **3. Complete periodic reanalysis of reported variants and exome data; consider additional genetic testing (e.g., genome sequencing, *FMR1* CGG repeat analysis if not already done, cytogenetic testing, if warranted, based on ES/CMA results and clinical findings) and/or referral for medical genetics evaluation if indicated**.

In most cases of patients with ID, GDD, and/or ASD, developmental, medical, and family histories do not point to a specific genetic etiology. In these cases, the approach to genetic testing should include broad analysis for copy number and exonic sequence variants (Table 4). This can be achieved by ordering a single genetic test - ES with CNV analysis (65). Diagnostic yields of ES and emerging evidence that ES is cost-efficient in the etiologic evaluation of children with neurodevelopmental disorders (142, 160–163) support this assertion that ES is the most appropriate first test to pursue (65). Payers are even altering their policies to support increasing efficiency through such testing algorithms (234).

It is important to note that, at present, some genetic testing laboratories may not include CNV analysis as part of their ES and some payers may require completion of CMA prior to coverage of ES. As a result, ES (without CNV analysis) and CMA may need to be pursued in a stepwise manner (65, 141). In cases where both CMA and ES are pursued independently, the order of testing will be dictated by individual factors such as insurance requirements (e.g., payer may only cover ES after a negative CMA) and additional phenotypic features (e.g., non-specific epilepsy phenotype in addition to ASD increasing the likelihood of a sequence variant) (235).

Less often, a patient presenting with ID, GDD, and/or ASD may have a constellation of features and/or history that are suggestive of a particular genetic etiology. In these instances, if a specific disorder is suspected, specific genetic evaluation(s) should be pursued (Table 4). For example, in a male patient with developmental delay, head circumference that is more than 2.5 standard deviations above the mean for age, and penile freckling, *PTEN* gene analysis may be pursued as an initial evaluation given that these features are highly suggestive of *PTEN* hamartoma tumor syndrome (13, 30). Similarly, in the case of a female with severe developmental delay, acquired microcephaly, seizures, and stereotypic hand movements, *MECP2* gene analysis may be pursued given that these features are suggestive of Rett syndrome (13, 79).

Recommendations for when to pursue *FMR1* repeat analysis are diverse with some publications recommending that all individuals with ASD and/or ID/GDD undergo testing (13, 14, 213) while others make a distinction based on sex and recommend testing for all males (54). Finally, some authors suggest ordering *FMR1* repeat analysis as a second-tier test (209) or restricting testing to those with the highest likelihood of testing positive for an *FMR1* full mutation based on specific clinical findings could reduce the number of individuals tested while maintaining high sensitivity (236–238). With the relatively low cost of *FMR1* repeat analysis and reproductive risks for family members should a child have fragile X, there should be a low threshold for offering this testing if testing is not offered to all patients with ID, GDD. and/or ASD.

Once a testing approach is determined, the ordering provider should engage families in a conversation prior to testing so they understand the nature and scope of the test(s) (e.g., purpose and potential results including uncertain results), benefits, limitations (e.g., does not detect all genetic causes of neurodevelopmental disorders), risks (e.g., uncertain results and familial implications), and costs (228, 233). Following receipt of results, providers will need to review any variants identified and put them in the context of their patient's clinical features and what is known about the gene, communicate results to the family, establish a follow-up plan, and collaborate with and refer to subspecialists (e.g., medical geneticist) as needed (233).

### Integrate a Genetic Counselor Into Your Clinic

Some non-genetics providers express a lack of knowledge and/or discomfort in ordering and interpreting genetic testing (239–243), but genomics education (244), relationships with genetic testing laboratory staff (245), and the ability to refer to genetics (241) once results are received can afford providers with knowledge and support to integrate genomic medicine into their practice. In addition to consenting for and ordering testing independently, pediatric providers can integrate genetic counselors into their clinics for additional support and expertise and a multidisciplinary approach to care. Genetic counselors provide care in at least 28 countries and are typically graduate level providers that have training in both genetics and counseling enabling them to interpret genetic test results and support patients and families in their care (246–249). Increasingly, genetic counselors are working as experts in genetic testing alongside non-genetics providers in various clinical settings, including pediatric neurology and neurodevelopmental clinics (250–252). With the ability to foster and maintain interdisciplinary relationships included in their code of ethics and practice-based competencies, genetic counselors can educate other providers about genetic testing and the genetics of neurodevelopmental disorders (249, 253–256). Furthermore, genetic counselors' expertise makes them well-suited to work alongside pediatric providers to obtain consent for genetic testing, aid in ordering of appropriate testing, interpret genetic test results, and facilitate a family's understanding of and adjustment to genetic information (179, 249, 253–259). Genetic counselors' skill sets can enable them to be important members of a multidisciplinary neurodevelopmental care team along with other specialists such as neurodevelopmental pediatricians (16).

### Refer to Genetics Provider

Pediatric healthcare providers may also elect to refer a subset of patients with neurodevelopmental disorders on to an external genetics provider (260). Medical geneticists have expertise in genetic diagnostics, management of genetic conditions, and counseling services (261). Pediatric healthcare providers might choose to refer to a genetics provider in cases where a specific genetic condition is suspected, additional etiological work-up is indicated after non-diagnostic testing, the provider is not equipped to facilitate discussion and ordering of specific testing, or where insurance requires a genetics evaluation before coverage of genetic testing (137, 234). Following receipt of results, pediatric healthcare providers could also refer to medical geneticists given that medical geneticists are well-poised to facilitate clinical correlation of genomic variants, evaluation of VUS, and management of patients with genetic diagnoses requiring ongoing surveillance and care (14, 137, 224, 262, 263).

### Approach to Genetic Testing Around the Globe

Given the diagnostic yields and implications of genetic test results, genetic testing should be considered for any patient with ID, GDD, or ASD across the globe (Table 4). With that said, approaches to testing might be influenced by country and/or regional-specific factors. The healthcare system (universal health coverage vs. primarily private, for profit insurers) (216), recognition of genetic professionals (223, 247), access to testing due to availability and cost (247, 264–266), and genetics education for non-genetics professionals (265–267) all affect integration of genetics into the care of patients with neurodevelopmental disorders.

## Additional Considerations for Today and the Future

Although the majority of the literature concerning genetic testing for patients with neurodevelopmental disorders focuses on genetic testing for three neurodevelopmental conditions – ASD, ID, and GDD; the rapid evolution of genomic medicine and expansion of the literature will continue to change the landscape of genetic testing shifting who is offered genetic testing, the test offerings available, and our understanding of genomic variants.

### Expanded Indications for Clinical Testing

In addition to ASD, ID, and GDD, increasing evidence supports consideration of genetic testing for other neurodevelopmental disorders. In epilepsy, between 4 and 78% of selected patients have genomic variants that are probable or definitive explanations for their epilepsy phenotypes (235). In a recent meta-analysis, of studies reporting on the yield of CMA, epilepsy gene panels, and ES, the diagnostic yield was 8, 23, and 45%, respectively (235). Specific epilepsy phenotypes, such as epileptic encephalopathies and comorbid ID have been associated with increased yields of testing (268, 269). Importantly, an increasing number of genetic conditions associated with seizure phenotypes influence therapeutic decision making; therefore, identification of a genetic etiology increasingly can enable personalized care (270–274). Despite genetic testing yields similar to those if ID, GDD, and ASD, no formal practice guidelines regarding genetic testing for individuals with epilepsy have been published by major medical societies; although various approaches to etiologic genetic testing in this patient population have been suggested by independent authors (273, 275).

In CP, studies have suggested a yield of 9.6–31.0% from CMA (276–279) and 10.6–65.0% using ES (280–283). In addition to emerging evidence that CP has a sizable genetic underpinning, genetic testing in CP has already resulted in changes to care (282, 284). Although a practice parameter published more than 15 years ago, the 2004 joint practice parameter from the American Academy of Neurology and Child Neurology Society, indicates that genetic testing should not be pursued in children with CP (285), no guidelines have been published since the emergence of data that demonstrates a significant diagnostic yield of genetic testing in CP patients.

Additional neurodevelopmental disorders, including speech disorders (286–290), Tourette syndrome (291–294) ADHD (295–298), and schizophrenia (299) have emerging evidence that genetic testing can provide an etiology to a portion of patients. Further studies are needed to understand the diagnostic yields and inform testing algorithms for these conditions.

The overlapping symptoms (300, 301), shared genetic underpinnings (4, 81, 211, 229, 302), and comorbidity of neurodevelopmental and neuropsychiatric diagnoses (303–307), have prompted many to advocate that neurodevelopmental and neuropsychiatric conditions collectively be considered as a continuum of developmental brain dysfunction (4, 229, 308). Familial aggregation and genetic studies have further supported the heterogenous nature of neurodevelopment and neuropsychiatric disorders (309–314). Given this and the emerging evidence of utility of genetic testing in a number of neurodevelopmental disorders, in the future, evidence may support broad implementation of genetic testing across neurodevelopmental and neuropsychiatric conditions.

### Advances in Clinical Testing

In addition to considering genetic testing for additional neurodevelopmental diagnoses, current testing technology will continue to improve, and new testing offerings will emerge. For example, ES analysis did not routinely detect copy number variants and uniparental disomy, but now some laboratories are identifying and reporting these potentially causative variants (121, 140). With the rapid improvement of testing technologies and bioinformatics pipelines, ES may one day be able to detect additional genetic causes of neurodevelopmental disorders, including trinucleotide repeat expansions (315).

Not only will existing testing continue to improve, the clinical integration of other testing modalities such as genome sequencing, that increases coverage of exons, improves detection of structural variants, and interrogates non-coding regions; RNA-sequencing that analyzes gene expression and mRNA splicing; and genome-wide methylation studies that assesses epigenetic modification of the genome that can impact gene expression will increase diagnostic yield (212, 316–319). It is estimated that up to 3% of all patients with negative exomes could be explained by variants in the non-coding, regulatory regions that would be detectable by genome sequencing (316) and could be further assessed through technologies such as RNA-sequencing (317) and genome-wide methylation studies (319, 320). In addition to informing pathogenicity of non-coding variants, RNA-sequencing and genome-wide methylation studies could also detect epigenetic changes that impact gene expression but do not alter the DNA sequence and are thus undetectable through sequencing (319, 321).

Furthermore, genome sequencing and single-cell sequencing will also enable increased detection of somatic mosaicism that, using current technologies, appear to constitute 5–10% of *de novo* variants (211, 322). Because somatic variants are not always detectable in the blood, future testing that includes additional cell types will also increase the identification of genetic etiologies for patients and families (211). Availability of relevant tissues, such as single neurons, may be limited, however.

In addition to advancements in testing technology, the understanding of genes and variants and their relationships to disease will continue to improve. Currently, only a fraction of the 20,000 human coding genes have an established disease relationship, and variants in known disease genes may be uncertain given the current knowledge base. As reference databases are bolstered (317), data sharing of variants and candidate genes increases (317, 323, 324), and additional technologies such RNA-sequencing and genome-wide methylation studies are integrated into testing algorithms (317, 319), our understanding of genes' relationship to neurodevelopment disorders and variants' pathogenicity will improve, thus increasing diagnostic yield of testing.

As new tests emerge and previous test results are reanalyzed or updated, recontacting of patients should be a shared responsibility among health-care providers, the clinical testing laboratories, and the patients (325, 326). As such, pediatric providers ordering genetic testing and those with an ongoing relationship with the patient and family, should remain informed of advancements that might provide additional information about a reported variant or indicate that additional testing or evaluation is warranted (82, 325, 327). Providers should bear in mind that, even if an individual has had genetic testing, there may be additional testing (e.g., exome reanalysis, CMA if ES does not include adequate CNV detection, genome sequencing, and novel testing such as genome sequencing) to consider over time and it is important that clinicians continue to consider the utility of genetic testing in individuals with neurodevelopmental diagnoses.

### Additional Genetic Factors

Currently, diagnostic genetic testing in neurodevelopmental disorders is focused on identifying rare variants of large effect size. As our ability to detect and interpret genetic causes of NDD increases, including those of more modest effect size, more families will be afforded the clinical and personal utility of genetic diagnoses. Many genetic underpinnings of NDD are characterized by variable expressivity, however, often leaving families with questions about recurrence and prognosis. In addition to the important impact of environmental and stochastic variation, recent advances suggest that the observed variability in phenotypic expression of large-effect rare variants may be explained, in part, by the additive effects of additional high-impact variants (328, 329) and background polygenic risk conferred by a large number of common variants of small individual effect size (330–336). Larger sample sizes and improved study methodologies are expected to lead to identification of rare variants of smaller effect size and elucidate the potential clinical role for polygenic scores in the near future. Increased understanding of additional genetic and other factors that influence phenotypes will enable tailored counseling and care for families receiving a genetic etiology for their child's NDD (17, 334).

## Conclusions

Neurodevelopmental disorders are common, and a significant proportion are caused by rare copy number and exonic sequence variants of large effect size that can be identified by genetic testing. Identifying a genetic etiologic diagnosis can allow clinicians to provide more accurate prognostication and recurrence risk counseling, identify and treat or prevent medical comorbidities, guide patients and families to condition-specific resources and supports and, in some cases, refine treatment options. Genetic testing is now the standard of care for individuals with ID/GDD and/or ASD, and the indications for testing will almost certainly broaden to include other neurodevelopmental disorders in the future. Most genetic etiologic diagnoses in patients with ID/GDD and/or ASD can be made with broad examination of copy number and exonic sequence variants that is most efficiently achieved using ES with CNV calling. It is important for clinicians who provide healthcare to children and adolescents with neurodevelopmental disorders to gain an understanding of these common tests and their role in providing the best medical care for patients and to work to facilitate the genetic etiologic evaluation of these patients by ordering testing or partnering with genetic providers.

## Author Contributions

JS and SM contributed to the literature review and synthesis of this review article.

## Conflict of Interest

The authors declare that the research was conducted in the absence of any commercial or financial relationships that could be construed as a potential conflict of interest.
